# Prognostic Value of Plasma N-terminal Pro-B-Type Natriuretic Peptide (Nt-proBNP) Levels in Patients Presenting With Cardiac Arrhythmias to the Emergency Department: A Prospective Observational Study From India

**DOI:** 10.7759/cureus.110665

**Published:** 2026-06-11

**Authors:** Shiwangini Jaiswal, Amit Rohila, Ankur Sharma, Mahaveer Singh Rodha, Rahul Choudhary, Bharat Choudhary, Siddhi Chawla

**Affiliations:** 1 Trauma and Emergency, All India Institute of Medical Sciences, Jodhpur, IND; 2 Cardiology, All India Institute of Medical Sciences, Jodhpur, IND

**Keywords:** arrhythmia, heart failure, nt-probnp, recurrence, sepsis

## Abstract

Introduction and rationale: N-terminal pro-B-type natriuretic peptide (NT-proBNP), a key marker of cardiac stress, is widely used in heart failure care. However, its role in patients presenting to the Emergency Department (ED) with cardiac arrhythmias remains underexplored.

Aims and objectives: To evaluate the prognostic significance of plasma NT-proBNP levels in patients presenting to the ED with cardiac arrhythmias and to assess their clinical profile.

Methodology: A prospective observational study was conducted over 18 months, from September 2023 to December 2024, with data analysis performed from January 2025 to May 2025, in the Department of Trauma and Emergency, All India Institute of Medical Sciences (AIIMS) Jodhpur, India. Patients aged ≥18 years with various arrhythmias were enrolled, excluding those with pre-existing heart failure, renal dysfunction, or structural heart disease. NT-proBNP levels, echocardiographic findings, clinical symptoms, and laboratory parameters were recorded and correlated with immediate outcomes (rhythm reversion versus recurrence). Arrhythmia recurrence was defined as the recurrence of the same or another clinically significant arrhythmia during hospitalization or within a seven-day follow-up period.

Results: In 194 patients with cardiac arrhythmias, 35.6% achieved rhythm reversion, while 64.4% experienced recurrence. NT-proBNP levels were significantly higher in the recurrence group (8545.53 vs. 2555.06 pg/mL, p=0.001), consistent across all left ventricular ejection fraction (LVEF) categories. Elevated NT-proBNP, sepsis, high-sensitivity C-reactive protein (hs-CRP), and low hemoglobin predicted poorer outcomes in univariable analyses, with septic shock showing the strongest adverse prognostic impact (p=0.015).

Conclusion: Elevated NT-proBNP levels were associated with an increased risk of arrhythmia recurrence and adverse short-term outcomes in patients presenting with cardiac arrhythmias. These associations were observed across different LVEF categories, suggesting that NT-proBNP may reflect underlying cardiac stress and overall illness severity. Sepsis and inflammatory states were also associated with poorer outcomes, highlighting the multifactorial nature of risk in this population. NT-proBNP may have utility as a risk stratification marker in the ED; however, larger prospective studies with multivariable adjustment are required to validate these findings and determine its independent prognostic value.

## Introduction

Cardiac arrhythmias, ranging from tachyarrhythmias such as atrial fibrillation (AF) to bradyarrhythmias, are common causes of Emergency Department (ED) visits and are associated with serious complications like stroke, heart failure, and sudden death [1]. The incidence of arrhythmias increases with advancing age and comorbidities like hypertension, diabetes, and chronic kidney disease [2-4]. As the Indian population ages, the burden of arrhythmia cases is expected to rise, particularly in resource-limited EDs. AF remains the most frequently encountered arrhythmia in emergency settings [5]. In India, AF may be underdiagnosed, with studies suggesting a prevalence of up to 17% among adults using ambulatory electrocardiography (ECG) monitoring [6]. Given the high prevalence of risk factors such as diabetes and hypertension in the Indian population, early identification of high-risk patients becomes crucial.

N-terminal pro-B-type natriuretic peptide (NT-proBNP), a peptide secreted in response to myocardial stretch, is an established biomarker for heart failure [7]. However, emerging evidence shows its levels also rise in patients with arrhythmias, independent of heart failure [8]. Elevated NT-proBNP in patients with new-onset AF has been associated with lower rates of successful cardioversion and worse long-term outcomes [5,9]. Importantly, even in those without overt heart failure, higher NT-proBNP levels predicted adverse events such as stroke and hospitalization [10]. NT-proBNP is released in response to myocardial wall stretch, pressure overload, neurohormonal activation, and inflammatory stress. Acute arrhythmias may promote atrial and ventricular remodeling, transient hemodynamic compromise, and myocardial stress, resulting in elevated NT-proBNP concentrations. Therefore, NT-proBNP may reflect both the severity of the arrhythmia episode and the underlying vulnerability to adverse outcomes.

The utility of NT-proBNP extends beyond AF. In patients with bradyarrhythmias and conduction blocks, increased NT-proBNP levels reflect hemodynamic stress due to atrioventricular (AV) dissociation [8]. Similarly, in systemic sclerosis, elevated NT-proBNP was associated with ventricular ectopy, suggesting it may serve as a marker of subclinical cardiac involvement [7].

In summary, NT-proBNP is a promising biomarker reflecting cardiac strain in a range of arrhythmias. Despite its potential, data on its prognostic utility in the Indian ED setting remain sparse. This study aims to evaluate whether NT-proBNP levels measured at ED presentation can help stratify short-term outcomes in patients with arrhythmias, while also examining the clinical and demographic characteristics of this population. Most previous studies have focused on individual arrhythmia subtypes, particularly AF. NT-proBNP may provide prognostic information beyond conventional clinical assessment by serving as an objective biomarker of myocardial stress, neurohormonal activation, and underlying cardiovascular vulnerability. Data evaluating NT-proBNP across a broader ED arrhythmia population remains limited, especially in India.

## Materials and methods

This prospective observational study was conducted in the Department of Trauma and Emergency Medicine, All India Institute of Medical Sciences (AIIMS) Jodhpur, India, over a period of 18 months from September 2023 to December 2024, with data analysis from January 2025 to May 2025. The study included 194 adult patients (aged ≥18 years) presenting to the ED with documented cardiac arrhythmias, identified via ECG. Although the estimated sample size, based on the ED census and symptom prevalence, was around 180, a total of 194 patients were enrolled due to variability in patient flow. The estimated sample size of approximately 180 patients was based on the expected prevalence of cardiac arrhythmia presentations to the ED during the study period and the projected number of eligible patients meeting inclusion criteria. Because of the exploratory nature of this prospective observational study and the limited availability of prior data regarding NT-proBNP-based prognostic assessment across heterogeneous arrhythmia types in the ED setting, a formal effect-size-based calculation could not be performed. Inclusion criteria encompassed all forms of bradyarrhythmias and tachyarrhythmias, while patients with heart failure, renal dysfunction, or valvular/structural heart disease were excluded.

Upon presentation, patients underwent structured clinical assessments, including primary and secondary surveys, an ECG for arrhythmia classification, and bedside transthoracic echocardiography to evaluate the LVEF. Demographic and clinical data, such as comorbidities (e.g., hypertension and diabetes), substance use, medications, and presenting symptoms, were systematically recorded. Alongside standard laboratory investigations, plasma NT-proBNP levels were measured by chemiluminescence immunoassay (CLIA) in the central biochemistry laboratory. Patients with a documented prior diagnosis of heart failure were excluded. Structural heart disease was excluded based on clinical history and echocardiographic assessment, including significant valvular heart disease, cardiomyopathy, or major structural abnormalities. Renal dysfunction was excluded based on clinical history, baseline laboratory evaluation (including serum creatinine), and available medical records. Despite these exclusions, occult subclinical cardiac or renal dysfunction could not be completely excluded in an emergency setting and may have contributed to NT-proBNP variability.

The primary outcome of the study was arrhythmia recurrence within seven days of index ED presentation. Patients were followed either through in-person visits or structured telephonic follow-up at seven days. Secondary outcomes were rhythm reversion, hospitalization, and mortality. Recurrence was defined as the reappearance of the same or clinically significant arrhythmia documented by ECG during hospitalization or reported during follow-up and confirmed from medical records within seven days of index presentation, when available. Rhythm reversion was defined as successful restoration of sinus rhythm or resolution of the presenting arrhythmia following initial management in the ED. Patients were grouped according to the presenting arrhythmia (e.g., AF/flutter, supraventricular tachycardia, ventricular arrhythmias, and bradyarrhythmias/conduction disorders).

Treatment decisions were made based on clinical judgment and standard hospital protocols, independent of NT-proBNP levels to avoid bias. Immediate outcomes such as arrhythmia recurrence and rhythm reversion were documented, and patients were followed up at seven days, either in person or telephonically, to assess for recurrence of arrhythmia, further need for hospitalization, or mortality. The study aimed to assess the prognostic role of NT-proBNP in predicting short-term clinical outcomes in emergency patients presenting with arrhythmias. Data were compiled in Microsoft Excel (Microsoft Corp., Redmond, WA, USA) and analyzed using SPSS version 26 (IBM Corp., Armonk, New York, USA). Continuous variables were presented as mean ± SD and compared using independent t-tests. Categorical data were analyzed using Chi-square tests. Analysis of variance (ANOVA) and receiver operating characteristic (ROC) curve analyses were performed for subgroup comparisons. A p-value <0.05 was considered statistically significant. NT-proBNP cutoff values were derived using ROC analysis with the Youden index to optimize sensitivity and specificity. Data distribution was assessed before analysis. Multivariable regression was not performed because the study was exploratory in nature.

## Results

The demographic details of the 194 patients were compared between the rhythm reversion and rhythm recurrence groups after initial management, as shown in Table 1. All demographic parameters were comparable, with no statistically significant differences between the groups.

**Table 1:** Baseline clinical characteristics and demographic details ^^^Chi-square test or Fisher's exact test. ^*^Student's t-test or independent samples t-test. CVA, cerebrovascular accident; SOB, shortness of breath; BP, blood pressure; NS, not significant

Parameter	Category	Reverted	Recurrence	Test value	P-value	Significance
Age	Mean±SD	56.71±16.73	60.26±17.24	1.389^*^	p=0.166	NS
Gender	Female	25 (28.1%)	64 (71.9%)	3.78^^^	p=0.055	NS
	Male	44 (41.9%)	61 (58.1%)			
BP (mmHg)	≥90/60	58 (36.7%)	100 (63.3%)	0.484^^^	p=0.486	NS
	<90/60	11 (30.6%)	25 (69.4%)			
Systolic BP	Mean±SD	119.67±29.71	122.37±32.46	0.561^*^	p=0.576	NS
Diastolic BP	Mean±SD	72.78±20.89	72.99±21.32	0.067^*^	p=0.947	NS
Heart rate	Mean±SD	143.99±63.44	139.38±60.23	0.501^*^	p=0.617	NS
Smoking	Yes	18 (42.9%)	24 (57.1%)	1.243^^^	p=0.265	NS
Alcohol	Yes	8 (38.1%)	13 (61.9%)	0.066^^^	p=0.789	NS
Opium	Yes	6 (31.6%)	13 (68.4%)	0.146^^^	p=0.702	NS
Tobacco	Yes	9 (50.0%)	9 (50.0%)	1.804^^^	p=0.179	NS
Symptoms	Chest pain (yes)	29 (47.5%)	32 (52.5%)	5.567^^^	p=0.018	S
	SOB (yes)	11 (18.6%)	48 (81.4%)	10.595^^^	p=0.001	S
	Palpitation (yes)	29 (46.0%)	34 (54.0%)	4.458^^^	p=0.035	S
	Diaphoresis (yes)	5 (41.7%)	7 (58.3%)	1.804^^^	p=0179	NS
	Altered sensorium (yes)	4 (50%)	4(50%)	0.758^^^	p=0.384	NS
	Vomiting (yes)	1 (20%)	4 (80%)	0.543^^^	p=0.461	NS
	Vertigo (yes)	1 (50%)	1 (50%)	0.184^^^	p=0.668	NS
	Unresponsive (yes)	0 (0%)	6 (100%)	3.418^^^	p=0.065	NS
	Fever (yes)	1 (11.1%)	8 (88.9%)	2.463^^^	p=0.117	NS
	Syncope (yes)	0 (0%)	4 (100%)	2.254^^^	p=0.133	NS
Complications	Sepsis (yes)	8 (20.0%)	32 (80.0%)	5.328^^^	p=0.021	S
	Septic shock (yes)	1 (6.7%)	14 (93.3%)	5.925^^^	p=0.015	S
	New onset event (yes)	24 (52.2%)	22 (47.8%)	7.256^^^	p=0.007	S
	Cardiogenic shock (yes)	17 (37.8%)	28 (62.2%)	0.125^^^	p=0.724	NS
	Hyperkalemia (yes)	2 (13.3%)	13 (86.7)	3.507^^^	p=0.061	NS
	Symptomatic bradycardia (yes)	3 (50%)	3 (50%)	0.563^^^	p=0.453	NS
	Pulmonary embolism (yes)	2 (40%)	3 (60%)	0.044^^^	p=0.834	NS
	Drug induced (yes)	4 (57.1%)	3 (42.9%)	1.475^^^	p=0.225	NS
	Cardioembolic CVA (yes)	3 (75%)	1 (25%)	2.771^^^	p=0.096	NS
	Hypertension emergency (yes)	0 (0%)	4(100%)	2.254^^^	p=0.133	NS
	Others (yes)	3 (25%)	9 (75%)	0.623^^^	p=0.430	NS

Among the other parameters, including symptoms at presentation, complications associated with arrhythmias, emergency interventions required, left ventricular ejection fraction (LVEF) on echocardiography, NT-proBNP levels, and other biochemical parameters, shortness of breath (SOB) (p=0.001), chest pain (p=0.018), and palpitations (p=0.035) were significantly more common in patients who experienced arrhythmia recurrence. Regarding complications, sepsis (p=0.021), septic shock (p=0.015), and new-onset cardiac events (p=0.007) were significantly associated with recurrence, highlighting the impact of systemic infection and acute cardiac instability on arrhythmia outcomes. Patients with sepsis- and septic shock-associated arrhythmias had higher recurrence rates, whereas those who presented with new-onset cardiac events had a higher likelihood of rhythm reversion with initial management. In terms of emergency interventions, patients requiring repeated dosing (p=0.001) and drug infusion (p=0.001) had higher recurrence rates, suggesting that greater pharmacological support reflects more severe or refractory arrhythmias. Similarly, unfavorable outcomes (p=0.007) were significantly linked to recurrence. Among biochemical parameters, elevated NT-proBNP (p=0.001), creatinine (p=0.027), high-sensitivity C-reactive protein (hs-CRP; p=0.016), and lower hemoglobin (p=0.037) were significantly associated with recurrence, suggesting the influence of myocardial strain, renal dysfunction, inflammation, and anemia. Radiographically, pulmonary edema (p=0.002) and lower respiratory tract infection (LRTI; p=0.003) were also significant predictors. Furthermore, hospital admission (p=0.001) and mortality (p=0.022) were both significantly higher among patients with recurrence, emphasizing the adverse prognostic implications of arrhythmia recurrence in this cohort (Tables 2, 3, 4).

**Table 2:** Therapeutic interventions in the ED ^^^Chi-square test or Fisher's exact test. ED, emergency department; NS, not significant; S, significant; TPI, temporary pacemaker insertion

Parameter	Category	Reverted	Recurrence	Test value	P-value	Significance
Emergency interventions	Pharmacological cardioversion (yes)	61 (34.9%)	114 (65.1)	0.393^^^	p=0.531	NS
	Electrical cardioversion (yes)	10 (33.3%)	20 (66.7%)	0.077^^^	p=0.781	NS
	TPI (yes)	5 (27.8%)	13 (72.2%)	0.525^^^	p=0.469	NS
Interventions	Repeated dosing (yes)	29 (21.3%)	107 (78.7%)	40.27^^^	p=0.001	S
	Infusion required (yes)	18 (19.8%)	73 (80.2%)	18.64^^^	p=0.001	S
	Outcome (yes)	5 (15.2%)	28 (84.8%)	7.232^^^	p=0.007	S

**Table 3:** Biochemical and laboratory parameters ^*^Student's t-test or independent samples t-test. NT-proBNP, N-terminal pro-B-type natriuretic peptide; Hb, hemoglobin; hs-CRP, high-sensitivity C-reactive protein

Parameter	Category	Reverted	Recurrence	Test value	P-value	Significance
NT-proBNP	N (mean±SD)	69 (2555.06±4169.92)	125 (8545.53±14948.65)	3.256^*^	p=0.001	S
Hb	N (mean±SD)	69 (12.72±2.32)	125 (11.90±2.75)	2.100^*^	p=0.037	S
Creatinine	N (mean±SD)	69 (1.18±0.84)	125 (1.69±1.77)	2.227^*^	p=0.027	S
hs-CRP	N (mean±SD)	69 (27.80±49.91)	125 (60.29±105.03)	2.421^*^	p=0.016	S

**Table 4:** Patient disposition and clinical outcome ^^^Chi-square test or Fisher's exact test.

Parameter	Category	Reverted	Recurrence	Test value	P-value	Significance
Hospital admission	Yes	33 (26.2%)	93 (73.8%)	13.791^^^	p=0.001	S
Death	Yes	6 (18.2%)	27 (81.8%)	5.244^^^	p=0.022	S

Across all LVEF categories, NT-proBNP levels were significantly higher in patients with arrhythmia recurrence compared to those who experienced rhythm reversion. Among patients with reduced LVEF (<40%), the mean NT-proBNP level was 9879.15 pg/mL in the recurrence group versus 2860.00 pg/mL in the reverted group (p=0.013). Similarly, in those with mildly reduced LVEF (40%-49%), recurrent cases had a mean NT-proBNP level of 9069.32 pg/mL compared to 2296.50 pg/mL in reverted patients (p=0.014). Even among patients with preserved LVEF (≥50%), NT-proBNP remained significantly elevated in those with recurrence (7350.62 pg/mL) versus those who reverted (2520.40 pg/mL, p=0.027).

These findings indicate that higher NT-proBNP levels are strongly associated with arrhythmia recurrence, and this association persisted across all LVEF categories, underscoring its possible prognostic utility across the full spectrum of EF. Elevated NT-proBNP thus reflects underlying myocardial stress and hemodynamic compromise that may predispose patients to recurrent arrhythmic events (Table 5).

**Table 5:** Distribution of NT-proBNP according to the immediate outcome among various ejection fraction classes ^*^Student's t-test or independent samples t-test. NT-proBNP, N-terminal pro-B-type natriuretic peptide; EF, ejection fraction

EF	Types	N	Mean NT-pro BNP	T-value	P-value
EF <40%	Reverted	11	2860.00	2.586	0.013^*^
	Recurrence	38	9879.15		
EF=40%-49%	Reverted	6	2296.50	2.579	0.014^*^
	Recurrence	31	9069.32		
EF >50%	Reverted	52	2520.40	2.255	0.027^*^
	Recurrence	56	7350.62		

ROC analysis of NT-proBNP as a predictor of arrhythmia recurrence demonstrated that its discriminative ability varied across different arrhythmia types, with several showing strong predictive performance (Table 6).

**Table 6:** AUC for recurrence among arrhythmia patients AF, atrial fibrillation; AUC, area under the curve; AV, atrioventricular; PSVT, paroxysmal supraventricular tachycardia; VF, ventricular fibrillation; VT, ventricular tachycardia

Arrythmia	Outcome	Area	Std. error	P-value	Asymptotic 95% confidence interval	
					Lower bound	Upper bound
AF	Recurrence	0.666	0.064	0.010	0.541	0.791
AV block	Recurrence	0.870	0.058	0.000	0.756	0.984
PSVT	Recurrence	0.674	0.092	0.074	0.494	0.855
VF	Recurrence	1.000	0.000	0.083	1.000	1.000
VT	Recurrence	0.731	0.125	0.114	0.487	0.975

**Figure 1:**
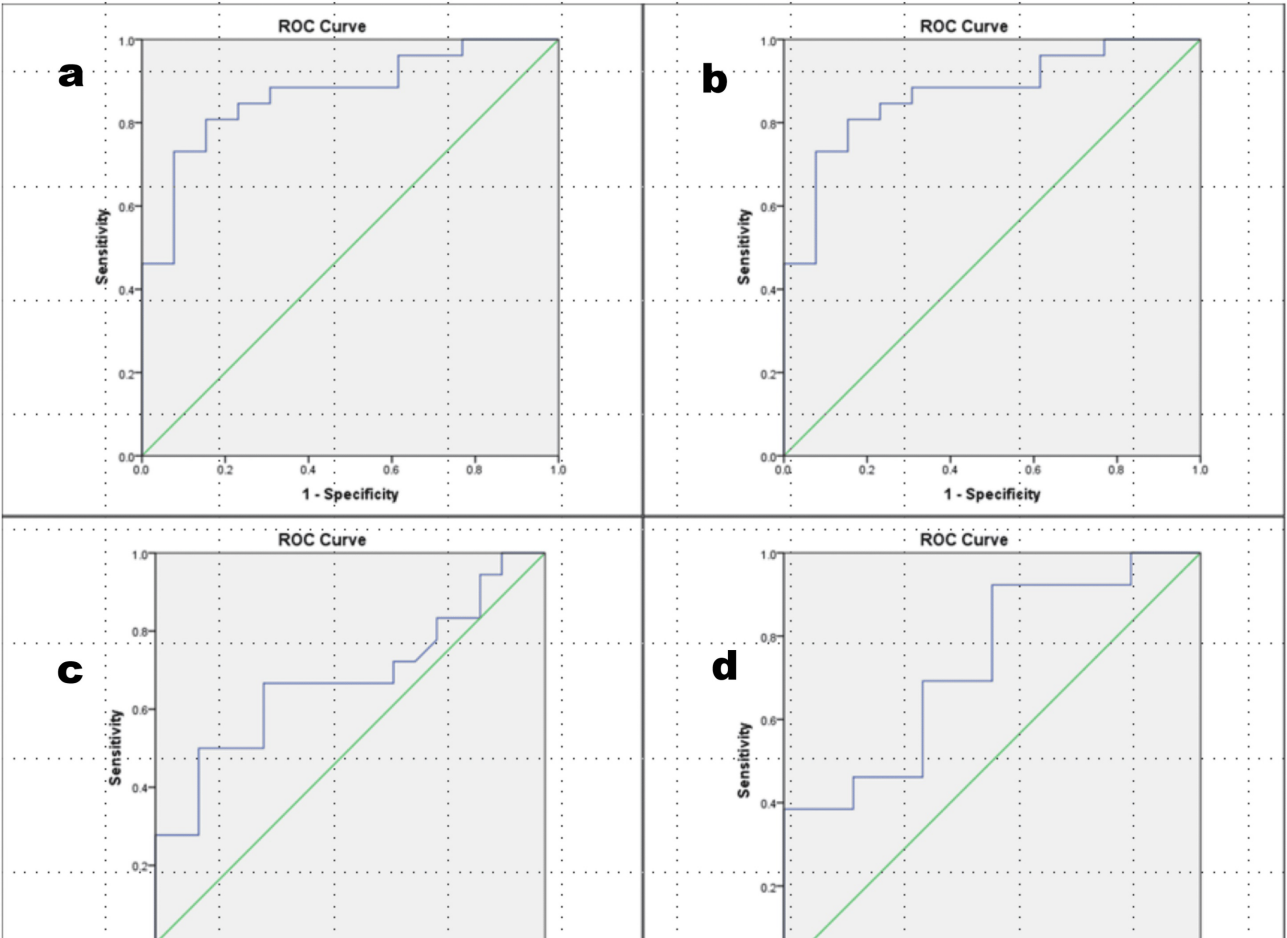
Showing ROC curve for AF (a), AV Block (b), PSVT (c), and VT (d) AF, atrial fibrillation; AV, atrioventricular; PSVT, paroxysmal supraventricular tachycardia; ROC, receiver operating characteristic

For AF, the area under the curve (AUC) was 0.666 (p=0.010), indicating a fair predictive value of NT-proBNP for recurrence. In AV block, the AUC was 0.870 (p<0.001), showing excellent discriminatory accuracy and suggesting that NT-proBNP is a strong predictor of recurrence in these patients. Among paroxysmal supraventricular tachycardia (PSVT) cases, the AUC was 0.674 (p=0.074), reflecting moderate predictive value but not reaching statistical significance. For ventricular fibrillation (VF), an AUC of 1.000 was observed, indicating perfect prediction, though the small sample size, as suggested by the high standard error and wide confidence interval, may limit interpretability. In ventricular tachycardia (VT), the AUC was 0.731 (p=0.114), suggesting good predictive ability, though again not statistically significant, possibly because of sample size limitations. In VF, ROC AUC 1.0 may be due to the small sample size. Overall, these results indicate that NT-proBNP shows a strong association with recurrence, particularly in AV block and AF, and a consistent trend toward association across other arrhythmia types, emphasizing its broad prognostic relevance in patients with arrhythmias (Figure 1). The wide confidence intervals of the ROC analyses warrant careful interpretation, as the quality of evidence is limited. ROC analyses for VF, VT, and PSVT should be interpreted cautiously because of small subgroup sample sizes. 

Table 7 summarizes the cutoff values, sensitivity, and specificity for predicting recurrence among patients with various arrhythmias.

**Table 7:** Cutoff values, sensitivity, and specificity for predicting recurrence in patients with various arrhythmias AF, atrial fibrillation; AV, atrioventricular; PSVT, paroxysmal supraventricular tachycardia; VF, ventricular fibrillation; VT, ventricular tachycardia

Arrythmias	Outcome	N	Cutoff value	Sensitivity	Specificity
AF	Recurrence	66	2090.00	71.2%	55.2%
AV block	Recurrence	26	1451.50	80.8%	76.9%
PSVT	Recurrence	18	432.00	66.7%	55.6%
VF	Recurrence	2	885.00	50.0%	0.0%
VT	Recurrence	13	2092.0	69.2%	66.7%

**Figure 2:**
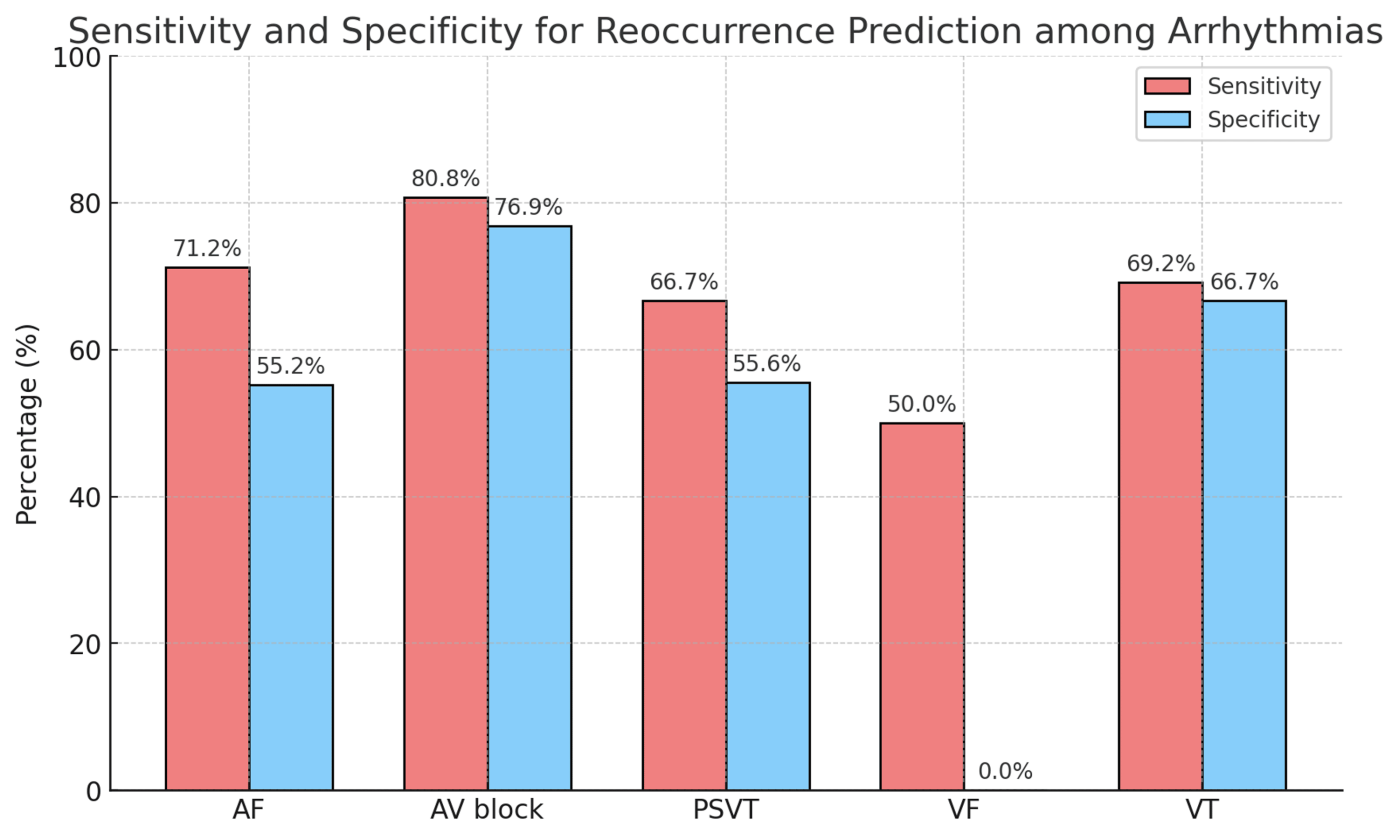
Sensitivity and specificity for predicting recurrence in patients with various arrhythmias AF, atrial fibrillation; AV, atrioventricular; PSVT, paroxysmal supraventricular tachycardia; VF, ventricular fibrillation; VT, ventricular tachycardia

For AF, among 66 patients, the cutoff value was 2090.00, with a sensitivity of 71.2% and a specificity of 55.2%. In patients with AV block (26 patients), the cutoff was 1451.50, yielding a sensitivity of 80.8% and a specificity of 76.9%, indicating strong predictive performance. For PSVT, among 18 patients, the cutoff value was 432.00, with a sensitivity of 66.7% and a specificity of 55.6%. In VF, which included only two patients, the cutoff value was 885.00, but it showed poor predictive capability, with a sensitivity of 50.0% and a specificity of 0.0%. Among 13 patients with VT, the cutoff value was 2,092.0, and the sensitivity and specificity were 69.2% and 66.7%, respectively. Overall, AV block showed the highest predictive accuracy based on both sensitivity and specificity (Figure 2).

## Discussion

This study underscores the prognostic relevance of NT-proBNP in patients presenting with cardiac arrhythmias to the ED. Beyond its established role in heart failure, NT-proBNP independently predicts arrhythmia recurrence, hospitalization risk, and in-hospital mortality, irrespective of LVEF. This indicates its utility in reflecting not just systolic dysfunction, but also atrial strain and systemic compromise, making it a valuable diagnostic and prognostic tool in acute care.

Elevated NT-proBNP levels are indicative of atrial wall stress during episodes of rapid or irregular rhythms like AF. This elevation reflects mechanical strain, atrial electrical dysfunction, and underlying structural changes such as atrial fibrosis [11]. Mehrabi et al. (2023) demonstrated a clear correlation between NT-proBNP levels and fibrosis severity in AF patients [12]. Moreover, NT-proBNP levels also respond to inflammatory activity. Trotter et al. (2021) found higher peptide levels in patients with systemic inflammation markers like interleukin-6 (IL-6) and tumor necrosis factor-alpha (TNF-α), supporting NT-proBNP’s role as a composite marker of mechanical, structural, and inflammatory stress [13]. In our study, particularly elevated NT-proBNP levels likely reflect the combined influence of all three. NT-proBNP's predictive superiority over other cardiac biomarkers stems from its longer half-life and its ability to reflect neurohormonal activation, including sustained renin-angiotensin-aldosterone and sympathetic nervous system activity. Unlike troponins or other natriuretic peptides, NT-proBNP provides a broader insight into the pathophysiological processes behind arrhythmias, including fibrosis, inflammation, and autonomic imbalance. In our study, AF recurrence was significantly associated with elevated NT-proBNP levels (p=0.001), with an AUC of 0.666 for differentiating recurrence. Additionally, patients with VT had significantly higher NT-proBNP levels than those with PSVT (p=0.004), suggesting more severe myocardial stress and potential structural heart disease. This is consistent with Muresan et al. (2017), who reported higher NT-proBNP levels correlating with ventricular arrhythmia burden, even in patients with preserved LVEF [7]. Similarly, Ocak et al. (2013) highlighted the utility of NT-proBNP in differentiating supraventricular from non-supraventricular tachycardias [14]. Our findings support and extend these observations by highlighting NT-proBNP’s role in risk stratification across arrhythmia types. Thus, elevated NT-proBNP levels may reflect acute myocardial stress, neurohormonal activation, sepsis-related myocardial dysfunction, transient renal impairment, and systemic inflammation rather than chronic heart failure alone.

N-terminal pro-B-type natriuretic peptide (NT-proBNP) and ejection fraction: clinical relevance, interpretation, and influencing factors

In this study, NT-proBNP emerged as a key biomarker for predicting adverse outcomes, including arrhythmia recurrence, prolonged hospitalization, and mortality, in patients presenting with arrhythmias. Notably, its prognostic utility remained consistent across all categories of LVEF, whether preserved (≥50%), mildly reduced (40%-49%), or reduced (<40%). This is particularly significant, as LVEF alone may not adequately reflect other critical factors such as myocardial wall stress, systemic inflammation, or neurohormonal activation.

Our findings suggest that NT-proBNP serves as an integrative marker of both cardiac and systemic stress, offering broader insight into patient status in acute settings. This is consistent with evidence from major studies and clinical guidelines. For example, Heart failure with preserved ejection fraction (HFpEF) clinical trials such as PARAGON-HF [15] incorporated NT-proBNP as part of the eligibility criteria, using higher thresholds in patients with AF to improve diagnostic specificity and risk stratification. For example, NT-proBNP thresholds of >200 pg/mL in sinus rhythm and >600 pg/mL in atrial fibrillation were used in selected patient groups. Similarly, Salah et al. (2019) demonstrated that NT-proBNP outperformed imaging in predicting outcomes in ambulatory HFpEF patients, with a caveat that levels may require adjustment based on body weight [16]. Further supporting these observations, Gómez-Otero et al. (2024) found that NT-proBNP levels exceeding 10,000 pg/mL at discharge in patients with heart failure with reduced ejection fraction (HFrEF) were significantly associated with poor recovery trajectories [17].

Collectively, these findings reinforce the conclusion that NT-proBNP provides meaningful prognostic information across the entire spectrum of ejection fractions. Its ability to capture underlying pathophysiological processes beyond systolic function makes it a valuable addition to clinical assessment. Accordingly, NT-proBNP may have a role in the risk stratification of emergency arrhythmia patients, irrespective of LVEF.

Role of N-terminal pro-B-type natriuretic peptide (NT-proBNP) levels in sepsis-associated recurrent cardiac arrhythmias

In our study, sepsis and septic shock emerged as significant clinical triggers for arrhythmia recurrence (p=0.015). This aligns with prior studies: Seemann et al. (2015) [18] reported a 42% incidence of new-onset supraventricular arrhythmias in septic shock, while Meierhenrich et al. (2015) found AF in 46% of patients with septic shock, closely linked to inflammatory markers like CRP [19]. Guenancia et al. (2014) also documented a 44% AF incidence, especially in patients with pre-existing cardiac dysfunction [20].

The arrhythmogenic environment in sepsis is multifactorial, driven by systemic inflammation, autonomic imbalance, catecholamine surge, electrolyte shifts, and myocardial stress. NT-proBNP may serve as a marker capturing these overlapping processes, helping identify high-risk patients early. Inflammatory cytokines like IL-6 and TNF-α alter cardiac conduction, while sympathetic overdrive and vagal suppression increase susceptibility [21,22]. Catecholamine excess impairs receptor responsiveness and worsens electrical instability; electrolyte and acid-base disturbances further destabilize cardiac rhythm [23-25].

Our data also found a significant association between anemia and arrhythmia recurrence (p=0.037). Prior studies support this. Negi et al. linked low hemoglobin with worse outcomes in acute coronary syndrome, while Hegde et al. observed arrhythmia reduction following anemia correction. In CKD patients, anemia was associated with LV hypertrophy and sudden cardiac death [26]. Anemia increases arrhythmic risk by reducing oxygen delivery and activating compensatory sympathetic responses.

AF was the most frequent arrhythmia (49%), and male patients were more likely to revert (p=0.045), consistent with findings by Swiss and multicenter studies indicating higher AF prevalence and better outcomes in men post-intervention [27,28].

Abnormal labs such as elevated creatinine and hs-CRP also correlated with poor prognosis. As per Jankiwaski et al. [29] and Smith et al. [30], elevated creatinine predicts cardiovascular mortality, while high hs-CRP has been linked to adverse outcomes in acute coronary syndrome (ACS), percutaneous coronary intervention (PCI), and stroke patients [31-35]. These biomarkers reflect systemic stress and organ dysfunction contributing to arrhythmic burden.

NT-proBNP, beyond its role in heart failure, showed strong potential as a prognostic tool in arrhythmic emergencies. Elevated levels were associated with recurrence, hospitalization, and mortality, irrespective of EF or arrhythmia type. For example, high NT-proBNP in AV block may prompt early pacing, while elevated levels in new-onset AF (mean 8545.53 pg/mL in the recurrence group) could indicate the need for prolonged monitoring [36]. In contrast, low NT-proBNP in stable PSVT may support early discharge.

Though the 2023 American College of Cardiology (ACC)/American Heart Association (AHA)/American College of Chest Physicians (ACCP)/Heart Rhythm Society (HRS) guidelines acknowledge BNP as a risk marker for AF, they do not recommend it for acute arrhythmia triage [37]. Our findings support further evaluation of its inclusion in ED-based risk models. Domenico et al. and Custodero et al. showed synergy between NT-proBNP and inflammatory markers like SII or hs-CRP, pointing to the potential of AI-driven composite risk scores.

NT-proBNP may have utility for risk stratification and warrants validation in larger studies. This study broadens NT-proBNP’s utility beyond heart failure, positioning it as an important biomarker for risk stratification in emergency arrhythmia care.

Limitation

This study has several limitations. First, the population was heterogeneous and included multiple arrhythmia subtypes. Second, multivariable adjustment was not performed, limiting the assessment of independent prognostic value. Third, some subgroup analyses were based on small sample sizes, particularly those involving ventricular arrhythmias. Fourth, NT-proBNP values demonstrated substantial variability and likely a non-normal distribution. Finally, residual confounding from acute illness severity, inflammation, and transient organ dysfunction cannot be excluded.

## Conclusions

This study highlights the prognostic value of NT-proBNP in patients presenting with cardiac arrhythmias to the ED. Elevated NT-proBNP levels were significantly associated with arrhythmia recurrence, hospitalization, and mortality. These findings suggest that NT-proBNP may serve as a useful marker of underlying cardiac stress and adverse short-term outcomes, even in patients without overt heart failure. The observed associations with hemoglobin, creatinine, and hs-CRP further highlight the relationship between NT-proBNP and overall illness severity. While NT-proBNP showed potential utility for risk stratification in this setting, the observational design and lack of multivariable adjustment preclude conclusions regarding independent prognostic value. Larger prospective studies with comprehensive adjustment for confounding factors, serial biomarker measurements, and longer follow-up are required to validate these findings and determine the clinical role of NT-proBNP in patients with cardiac arrhythmias.

## References

[1] Gaur U, Gadkari C, Pundkar A (2024). Associated factors and mortality of arrhythmia in emergency department: a narrative review. Cureus.

[2] Akhtar Z, Leung LW, Kontogiannis C, Chung I, Bin Waleed K, Gallagher MM (2022). Arrhythmias in chronic kidney disease. Eur Cardiol.

[3] Lip GY, Coca A, Kahan T (2017). Hypertension and cardiac arrhythmias: a consensus document from the European Heart Rhythm Association (EHRA) and ESC Council on hypertension, endorsed by the Heart Rhythm Society (HRS), Asia-Pacific Heart Rhythm Society (APHRS) and Sociedad Latinoamericana de Estimulación Cardíaca y Electrofisiología (SOLEACE). Europace.

[4] Grisanti LA (2018). Diabetes and arrhythmias: pathophysiology, mechanisms and therapeutic outcomes. Front Physiol.

[5] Diakantonis A, Verras C, Bezati S (2024). Predictive value of N-terminal pro B-type natriuretic peptide for short-term outcome of cardioversion in patients with first-diagnosed or paroxysmal atrial fibrillation presenting to the emergency department. Biomedicines.

[6] Rao MS, Mullasari A, Hiremath JS (2024). Prevalence of atrial fibrillation on a 24-hour Holter in adult Indians. Indian Heart J.

[7] Muresan L, Petcu A, Muresan C (2017). The role of NT-proBNP in the diagnosis of ventricular arrhythmias in patients with systemic sclerosis. Iran J Public Health.

[8] Pan W, Su Y, Hu K, Shu X, Ge J (2009). Effect of bradyarrhythmia on the plasma levels of N-terminal pro-brain natriuretic peptide. Int J Cardiol.

[9] Holl MJ, van den Bos EJ, van Domburg RT, Fouraux MA, Kofflard MJ (2018). NT-proBNP is associated with mortality and adverse cardiac events in patients with atrial fibrillation presenting to the emergency department. Clin Cardiol.

[10] Hamatani Y, Iguchi M, Ueno K (2021). Prognostic significance of natriuretic peptide levels in atrial fibrillation without heart failure. Heart.

[11] Zografos TA, Katritsis DG (2013). Natriuretic peptides as predictors of Atrial Fibrillation recurrences following electrical cardioversion. Arrhythm Electrophysiol Rev.

[12] Nasab Mehrabi E, Toupchi-Khosroshahi V, Athari SS (2023). Relationship of atrial fibrillation and N terminal pro brain natriuretic peptide in heart failure patients. ESC Heart Fail.

[13] Fish-Trotter H, Ferguson JF, Patel N (2020). Inflammation and circulating natriuretic peptide levels. Circ Heart Fail.

[14] Ocak T, Erdem A, Duran A (2013). The diagnostic significance of NT-proBNP and troponin I in emergency department patients presenting with palpitations. Clinics (Sao Paulo).

[15] Solomon SD, Rizkala AR, Gong J (2017). Angiotensin receptor neprilysin inhibition in heart failure with preserved ejection fraction: rationale and design of the PARAGON-HF trial. JACC Heart Fail.

[16] Salah K, Stienen S, Pinto YM (2019). Prognosis and NT-proBNP in heart failure patients with preserved versus reduced ejection fraction. Heart.

[17] López-Vilella R, Gómez-Otero I, Donoso Trenado V (2025). NT-proBNP in acute de novo heart failure: a key biomarker for predicting myocardial recovery-Comfe Registry. Life (Basel).

[18] Seemann A, Boissier F, Razazi K, Carteaux G, de Prost N, Brun-Buisson C, Mekontso Dessap A (2015). New-onset supraventricular arrhythmia during septic shock: prevalence, risk factors and prognosis. Ann Intensive Care.

[19] Meierhenrich R, Steinhilber E, Eggermann C (2010). Incidence and prognostic impact of new-onset atrial fibrillation in patients with septic shock: a prospective observational study. Crit Care.

[20] Guenancia C, Binquet C, Laurent G (2015). Incidence and predictors of new-onset atrial fibrillation in septic shock patients in a medical ICU: data from 7-day Holter ECG monitoring. PLoS One.

[21] Lazzerini PE, Abbate A, Boutjdir M, Capecchi PL (2023). Fir(e)ing the rhythm: inflammatory cytokines and cardiac arrhythmias. JACC Basic Transl Sci.

[22] Carrara M, Ferrario M, Bollen Pinto B, Herpain A (2026). The autonomic nervous system in septic shock and its role as a future therapeutic target: a narrative review. Ann Intensive Care.

[23] Najafi A, Sequeira V, Kuster DW, van der Velden J (2016). β-adrenergic receptor signalling and its functional consequences in the diseased heart. Eur J Clin Invest.

[24] Urso C, Brucculeri S, Caimi G (2015). Acid-base and electrolyte abnormalities in heart failure: pathophysiology and implications. Heart Fail Rev.

[25] Mitchell JH, Wildenthal K, Johnson RL Jr (1972). The effects of acid-base disturbances on cardiovascular and pulmonary function. Kidney Int.

[26] Hegde N, Rich MW, Gayomali C (2006). The cardiomyopathy of iron deficiency. Tex Heart Inst J.

[27] Samim D, Choffat D, Vollenweider P, Waeber G, Marques-Vidal P, Méan M (2023). Prevalence of atrial fibrillation : the Swiss population-based CoLaus|PsyCoLaus study. Herz.

[28] Noubiap JJ, Pathak RK, Thomas G, Elliott AD, Sanders P, Middeldorp ME (2024). Sex differences in outcomes of an intensive risk factor modification program in patients with atrial fibrillation. Circ Arrhythm Electrophysiol.

[29] Jankowski J, Floege J, Fliser D, Böhm M, Marx N (2021). Cardiovascular disease in chronic kidney disease: pathophysiological insights and therapeutic options. Circulation.

[30] Smith GL, Masoudi FA, Shlipak MG, Krumholz HM, Parikh CR (2008). Renal impairment predicts long-term mortality risk after acute myocardial infarction. J Am Soc Nephrol.

[31] Katamine M, Minami Y, Nagata T (2025). High-sensitivity C-reactive protein, plaque vulnerability and adverse events in patients with stable coronary disease: an optical coherence tomography study. Int J Cardiol.

[32] Prasanna R, Pasupathy S, Moidu F (2019). Etiology, clinical profile and outcome of first episode of seizure in children. Int J Contemp Pediatr.

[33] Hemmati R, Mohsenzadeh Y, Madadi R (2023). Association between the increased level of high-sensitive CRP (hs CRP) and non-arrhythmic ECG changes and echocardiographic abnormalities in patients with acute coronary syndrome. Caspian J Intern Med.

[34] Birrell H, Isles C, Fersia O, Anwar M, Mondoa C, McFadyen A (2024). Assessment of the diagnostic value of NT-proBNP in heart failure with preserved ejection fraction. Br J Cardiol.

[35] Kravitz BA, Corrada MM, Kawas CH (2009). High levels of serum C-reactive protein are associated with greater risk of all-cause mortality, but not dementia, in the oldest-old: results from The 90+ Study. J Am Geriatr Soc.

[36] Iwańska K, Gworys P, Gawor Z (2010). Prognostic value of NT-proBNP levels in patients undergoing permanent pacemaker implantation. Pol Arch Med Wewn.

[37] Joglar JA, Chung MK, Armbruster AL (2024). Correction to: 2023 ACC/AHA/ACCP/hrs guideline for the diagnosis and management of atrial fibrillation: a report of the American College of Cardiology/American Heart Association Joint Committee on clinical practice guidelines. Circulation.

